# Targeting NADPH oxidase-driven oxidative stress in diabetic cardiomyopathy: mechanisms and therapeutic perspectives

**DOI:** 10.3389/fphar.2025.1610429

**Published:** 2025-07-03

**Authors:** Zilv Ye, Sutong Wang, Zhengdong Wan, Birun Huang, Jiawei Guo

**Affiliations:** ^1^ Department of Vascular and Endovascular Surgery, The First Affiliated Hospital of Yangtze University, Jingzhou, Hubei, China; ^2^ Department of Pharmacology, School of Medicine, Yangtze University, Jingzhou, China; ^3^ Department of Laboratory Medicine, Jingzhou Hospital Affiliated to Yangtze University, Jingzhou, China

**Keywords:** diabetic cardiomyopathy, NADPH oxidase, reactive oxygen species, oxidative stress, redox signaling pathways

## Abstract

Diabetic cardiomyopathy (DCM) is a major complication of diabetes mellitus, characterized by microvascular dysfunction and progressive structural and functional deterioration of the heart. A central driver of DCM pathogenesis is chronic oxidative stress (OS), primarily resulting from excessive production of reactive oxygen species (ROS) under hyperglycemic conditions. Among the various ROS sources, the NADPH oxidase (NOX) family of enzymes plays a pivotal role in initiating and sustaining oxidative damage. NOX-mediated ROS production contributes to myocardial inflammation, apoptosis, fibrosis, and remodeling, through multiple signaling pathways, including NF-κB, TGF-β/Smad, MAPK, and PI3K/Akt cascades. Despite growing recognition of NOX enzymes as crucial mediators in DCM, therapeutic options specifically targeting NOX-driven oxidative stress remain limited. In this comprehensive review, we summarize current insights into the mechanisms by which NOX regulates cardiac pathology in DCM, highlight the crosstalk between NOX activity and downstream molecular pathways, and discuss potential pharmacological interventions aimed at restoring redox homeostasis. Emerging strategies, such as selective NOX inhibitors, antioxidant therapies, and agents modulating signaling transduction, offer promising avenues for mitigating oxidative injury and improving cardiac function. Furthermore, we emphasize the importance of developing isoform-specific NOX inhibitors to achieve greater efficacy and safety in clinical applications. By providing a detailed overview of NOX-dependent oxidative stress in DCM and associated therapeutic approaches, this review aims to foster further research and innovation toward targeted treatments for diabetic cardiomyopathy.

## 1 Introduction

DCM refers to structural and functional abnormalities of the heart observed in diabetic patients without coronary artery disease, hypertension, or other forms of heart disease (Zhao et al., 2022). Initially described in 1972, autopsy findings from four diabetic patients revealed unexplained cardiac enlargement and congestive heart failure, offering the first evidence of DCM (Rubler et al., 1972). As a prevalent chronic complication of diabetes, nearly all diabetic patients are at risk of developing cardiac issues, especially those with type 2 diabetes, who exhibit a higher incidence of DCM (Kenny and Abel, 2019). This condition is frequently associated with pathological processes, including oxidative stress, inflammation, and metabolic dysfunction (Marino et al., 2023). Importantly, DCM is usually asymptomatic in its early stages, complicating early diagnosis (Zhang L. et al., 2022). Clinical studies have shown that the myocardium of diabetic patients is more susceptible to damage from acute myocardial infarction and other injuries compared to non-diabetic individuals. Furthermore, diabetic patients exhibit diminished cardiac recovery capacity, leading to poorer rehabilitation outcomes (Shah et al., 2010; Pérez-Cremades et al., 2023). In the absence of timely detection and intervention, DCM can result in severe clinical consequences. Given the increasing global prevalence of diabetes, DCM has emerged as a critical component of diabetes-related cardiovascular diseases and a major contributor to the elevated risk of heart failure and mortality among diabetic patients (Kobayashi and Liang, 2015; Ma et al., 2023). Therefore, identifying effective therapeutic targets for the prevention and management of DCM is crucial.

OS is recognized as a key pathological mechanism in DCM and has emerged as a central focus of research. OS results from an imbalance between excessive ROS production and inadequate antioxidant defense capacity (Ritchie and Abel, 2020). The overproduction of ROS is considered a key mechanism underlying diabetes-induced inflammation and cardiac remodeling (Giacco and Brownlee, 2010; Zhao et al., 2023). Deficiencies in the antioxidant defense system exacerbate OS during the later stages of DCM, further promoting cardiac insulin resistance, disease progression, and heart failure (Zhou and Tian, 2018). Numerous studies suggest that modulating OS signaling pathways and reducing ROS production and accumulation offer promising therapeutic strategies for DCM (Peng et al., 2022; Tang et al., 2022). Accumulated ROS not only directly damage cardiomyocytes but also induce structural and functional alterations in the heart through various signaling pathways. Notably, the NOX family, the only enzymes specifically dedicated to ROS generation, plays a crucial role in the pathogenesis of DCM. NOX family members are transmembrane proteins that catalyze the reduction of oxygen to generate ROS, such as superoxide and hydrogen peroxide, in association with NADPH oxidation (Ogboo et al., 2022; Nabeebaccus et al., 2023). Increased NOX activity leads to excessive ROS production, exacerbating OS and accelerating the onset and progression of DCM.

This review investigates the biological mechanisms of NOX, the pathways through which NOX-mediated OS contributes to cardiac pathological changes, and recent advances in NOX-targeted therapies for DCM. We offer a comprehensive analysis of NOX-related signaling pathways in DCM and explore the potential of targeting NOX-mediated OS as a novel therapeutic approach.

## 2 NOX: a primary driver of oxidative stress

The development of DCM is strongly associated with oxidative stress, with members of the NOX family acting as key drivers of this process. NOX enzymes generate ROS, which in turn induce inflammation, cellular damage, and cardiac remodeling in heart tissue, thereby accelerating the progression of DCM. NOX activity is markedly elevated in diabetic patients (Daiber et al., 2020; Vilas-Boas et al., 2021), making it a critical factor in cardiac injury and functional deterioration.

### 2.1 The discovery and fundamental functions of NOX enzymes

The classical nicotinamide adenine dinucleotide phosphate (NADPH) oxidase was first identified in neutrophils in 1933, when Baldridge and Gerrard observed a significant increase in oxygen consumption during bacterial phagocytosis (Cross and Segal, 2004). It was later correctly identified in 1964 by Rossi and Zatti as the key enzyme responsible for initiating the respiratory burst (Rossi and Zatti, 1964). Through extensive research on the different subtypes of NOX, scientists have systematically characterized their structure and function within cells. In 1986, NOX2 was successfully cloned, marking a significant milestone in this field. Over the subsequent decades, other members of the NOX family, such as NOX1, NOX3, and NOX4, were also cloned using advanced molecular cloning techniques. NOX is expressed in phagocytic cells such as neutrophils, eosinophils, monocytes, and macrophages, which are critical components of the innate immune system. NOX is a multi-protein complex that catalyzes the reduction of oxygen and NADPH to produce superoxide (Kleniewska et al., 2012). It comprises two membrane-bound subunits (gp91phox and p22phox), three cytosolic subunits (p47phox, p67phox, and p40phox), and a small G protein (Rac1 or Rac2). In resting phagocytes, NOX remains inactive but becomes activated upon interaction with pathogens and subsequent engulfment by the phagosome (Touyz et al., 2019; Vermot et al., 2021). NOX is crucial for the oxidative stress response in cells, where it generates ROS using NADPH. In parallel, cells depend on reduced glutathione (GSH) as the primary antioxidant to neutralize these ROS. NADPH serves not only as the source of ROS production by NOX but also contributes to the reduction of oxidized glutathione disulfide (GSSH) to GSH through the enzyme glutathione reductase (GR), thereby sustaining the cell’s antioxidant defense. This process enables cells to manage the ROS produced by NOX, preserve redox homeostasis, and avoid the accumulation of oxidative damage (Li et al., 2023; Su et al., 2023).

### 2.2 Members of the NOX family and their activation mechanisms

The NOX family represents the primary regulated source of ROS formation, comprising seven isoforms with diverse tissue distribution and activation mechanisms: NOX1, NOX2, NOX3, NOX4, NOX5, and the dual oxidases Duox1 and Duox2 (Pecchillo Cimmino et al., 2023). The activation mechanisms of NOX subtypes vary, yet all NOX family members share common structural features, including six conserved transmembrane domains, four conserved heme-binding histidines, an FAD-binding domain, and an NADPH-binding domain (Begum et al., 2022). NOX enzymes transfer electrons from NADPH to the heme group, and subsequently to molecular oxygen, ultimately generating superoxide (O_2_^−^), a critical process in cellular oxidative stress. The activation of NOX family members typically requires small GTPases, such as Rac1, and several cytosolic subunits (Ogboo et al., 2022). NOX1 activation involves p22phox, RAC1, p47phox, and/or NOXO1, along with NOXA1. NOX2 activation requires p22phox, RAC1 or RAC2, p47phox, p67phox, and p40phox. NOX4 activation requires p22phox, and its activity can be modulated by POLDIP2. The activation of NOX5 is primarily regulated by Ca2+. DUOX1 and DUOX2 require both DUOXA1 and DUOXA2, along with Ca2+, for activation, and neither is expressed in the cardiovascular system (Zhang et al., 2020).

Their subcellular localization varies, ranging from the plasma membrane to intracellular compartments and the nuclear membrane (Pecchillo Cimmino et al., 2023). Among these, NOX2 and NOX4 are highly expressed in cardiomyocytes, endothelial cells, and fibroblasts (Cadenas, 2018; Jiang et al., 2020; Visnagri et al., 2023). NOX2 is a membrane-bound enzyme activated by stimuli such as angiotensin II (Ang-II), endothelin-1 (ET-1), TNF-α, growth factors, cytokines, and mechanical stress. In contrast, NOX4, located on intracellular membranes, exhibits constitutive activity (Sag et al., 2014; Moris et al., 2017; Bode et al., 2023; Nabeebaccus et al., 2023; Wang et al., 2023). NOX3 is highly expressed in different regions of the inner ear, such as the vestibule, cochlear sensory epithelium, and spiral ganglion, which led to its designation as inner ear NOX (Wang et al., 2023). Duox1 and Duox2 are exclusively expressed in epithelial cells (Brandes et al., 2010).

### 2.3 NOX-induced oxidative stress and its contribution to the pathogenesis of cardiovascular diseases

NOX is the only enzyme whose primary function is the generation of ROS. Its expression and activity in the heart play crucial roles in both physiological and pathological conditions. The ROS produced by NOX can trigger endothelial nitric oxide synthase (eNOS) uncoupling and mitochondrial dysfunction, leading to persistent oxidative stress (OS) and the development of cardiovascular diseases (CVD) (Zhang et al., 2020). NOX has emerged as a key oxidase system contributing to oxidative stress in vascular diseases such as hypertension, aortic aneurysm, hypercholesterolemia, atherosclerosis, and diabetic vascular complications, as well as in cardiovascular diseases including ischemia-reperfusion (IR) injury, myocardial infarction (MI), heart failure, and arrhythmias. Among the NOX family, NOX1, NOX2, and NOX4 are highly expressed in cardiac tissue. Previous studies have demonstrated that inhibition of NOX2 alleviates the harmful effects of hyperglycemia on cells, and that inhibiting NOX2-induced oxidative stress may prevent DCM (Tang et al., 2019; Zhu et al., 2024). NOX4 has been shown to reduce myocardial interstitial fibrosis in diabetic mice by regulating the Akt/mTOR and NF-κB pathways, leading to improved cardiac function (Zhao et al., 2015). NOX1 has been implicated in several cardiovascular diseases, particularly atherosclerosis, hypertension, and ischemia/reperfusion injury (Zhang D. et al., 2022). Increasing experimental evidence suggests that genetic modification of NOX isoforms exerts specific effects on cardiovascular phenotypes in animal models, emphasizing the central role of NOX in CVD progression (Zhou L. et al., 2021; Qiu et al., 2022; Yang et al., 2023; Huang et al., 2024).

In the context of hyperglycemia, NOX is further activated through specific metabolic pathways. Elevated blood glucose increases intracellular glucose levels, resulting in the accumulation of diacylglycerol (DAG), an intermediate in triglyceride synthesis. DAG functions as a critical signaling molecule that activates protein kinase C (PKC) (Khan, 2021). Activated PKC, in turn, stimulates NOX, promoting ROS production (Jubaidi et al., 2022). These ROS exacerbate OS, contributing to cellular damage and the development of cardiovascular and other complications. This signaling pathway has gained attention for its role in regulating angiogenesis, mitigating OS by reducing ROS production, and inducing cell death (Volpe et al., 2018).

### 2.4 NOX-induced oxidative stress and its impact on cardiac progenitor and stem cells (CPCs)

OS plays a crucial role in the dysfunction of CPCs, particularly in the context of cardiovascular diseases such as DCM (Chen et al., 2018). Excessive production of ROS leads to CPC damage, resulting in impaired proliferation, restricted differentiation potential, and accelerated aging (Molinaro et al., 2022). CPCs are essential for myocardial regeneration. However, under pathological conditions like DCM, their functionality is significantly compromised, limiting cardiac repair and exacerbating heart failure progression. Consequently, modulating oxidative stress to restore CPC function represents a promising therapeutic strategy (Chen et al., 2018).

In this regard, antioxidants, such as resveratrol, or redox modulators have been shown to reduce ROS accumulation, significantly enhancing the proliferative and differentiative capacities of CPCs, thereby promoting cardiac repair (Dudek et al., 2021). This approach not only decelerates the progression of heart failure but also opens new avenues for the clinical management of diabetes and other metabolic heart diseases.

Furthermore, NOX2 and NOX4 are critical in regulating CPC function. Research indicates that NOX2 generates ROS, which promotes angiogenesis and supports cardiac repair. ROS produced by NOX2 activate key factors, including hypoxia-inducible factor (HIF-1α) and vascular endothelial growth factor (VEGF), thereby enhancing angiogenesis and improving the cardiac repair microenvironment, which indirectly supports CPC-mediated repair (Urao and Ushio-Fukai, 2013). Additionally, NOX4 plays a pivotal role in CPC differentiation by modulating ROS levels, thus influencing their differentiation status (Nadworny et al., 2013).

These findings suggest that NOX2 and NOX4 are not only crucial for regulating CPC proliferation and differentiation but also may indirectly facilitate cardiac repair by influencing the cellular microenvironment. Therefore, targeting the OS pathways of NOX provides valuable insights into therapeutic strategies for restoring CPC function and promoting cardiac repair.

### 2.5 The potential role of NOX in the early diagnosis of DCM

In the early stages of DCM, NOX activity is markedly elevated, making it a potential biological marker for early diagnosis. NOX generates ROS, resulting in oxidative damage and inflammation in cardiomyocytes. These changes can be detected through blood tests for NOX-derived products, such as hydrogen peroxide and superoxide anion, or by directly measuring NOX expression levels to enable early identification (Yu et al., 2007; Nie et al., 2023). Furthermore, as diabetes progresses, the elevation in NOX activity is closely correlated with the severity of cardiac damage (Faria and Persaud, 2017; Maqbool et al., 2020), allowing for more accurate detection of early cardiac injury. Combining traditional cardiac biomarkers (such as BNP and cTnI) with NOX activity measurements can significantly improve the sensitivity and specificity of early diagnosis. By using multiple biomarkers, it is possible to more accurately assess myocardial injury in diabetic patients and detect early changes in heart function.

However, the early diagnosis still faces significant challenges due to the lack of specific biomarkers, subtle early symptoms, insufficient standardized detection methods, and difficulties in detecting early changes. Moreover, the absence of unified screening guidelines further complicates the situation. With advancements in technology and ongoing research, NOX and other biomarkers are expected to provide more precise and efficient methods for the early diagnosis of DCM in the future.

## 3 The involvement of NOX-regulated signaling pathways in the development and progression of DCM

As diabetes progresses, excessive activation of NOX induces oxidative stress in cardiac cells, which affects cardiac function and structure through multiple signaling pathways. The activation of these pathways not only promotes inflammation but also exacerbates apoptosis, fibrosis, and cardiac remodeling in cardiomyocytes, ultimately contributing to heart failure. Current research suggests that NOX-regulated signaling pathways play a multifaceted role in oxidative stress in DCM and are crucial for the progression of the disease.

### 3.1 Interaction between NOX and NF-κB signaling pathways

In DCM, NOX activation leads to the generation of ROS, which activates the NF-κB signaling pathway, crucial for regulating immune responses and inflammation. NOX-induced ROS enhances NF-κB activity (Zhang D. et al., 2022), and also activates signaling molecules like IKK and IκB through oxidative stress accumulation. This translocates NF-κB to the nucleus, promoting the expression of pro-inflammatory cytokines such as TNF-α, IL-1β, and IL-6, contributing to inflammation, cardiomyocyte damage, and dysfunction.

For example, NOX1 induces ROS accumulation and activates the TLR2/NF-κB pathway, promoting myocardial fibrosis in DCM (Zhang D. et al., 2022). Under hyperglycemic conditions, NOX-generated ROS enhances the expression of these pro-inflammatory cytokines, triggering inflammatory reactions that lead to cardiac injury and functional decline (Liang et al., 2018). Similarly, NOX4 activates NF-κB signaling through ROS, further exacerbating inflammation and fibrosis in DCM (Fan et al., 2018). Thus, NOX activation of the NF-κB pathway accelerates the progression of DCM, highlighting the potential for NOX-targeted therapies.

### 3.2 Interaction between NOX and TGF-β/Smad signaling pathway

The TGF-β/Smad signaling pathway plays a crucial role in the development of various cardiovascular diseases, particularly in cardiac fibrosis. NOX4 is key in DCM, where it activates the TGF-β/Smad signaling pathway through ROS generation, promoting the proliferation of cardiac fibroblasts and collagen deposition. ROS activate downstream Smad proteins by interacting with the TGF-β receptor, which leads to fibroblast transformation and worsens the fibrosis process.

For instance, NOX generates ROS, which activates the TGF-β/Smad signaling pathway and promotes myocardial fibrosis in DCM. The accumulation of ROS increases the expression of TGF-β, thereby further activating the Smad2/3 pathway, promoting fibroblast proliferation and collagen deposition, which exacerbates cardiac fibrosis and functional impairment (Fan et al., 2018). NOX activates the TGF-β/Smad pathway through ROS generation, while BCP (a compound) inhibits TGF-β signaling, indirectly reducing NOX-induced ROS production, thereby alleviating cardiac fibrosis and endothelial-mesenchymal transition (Hashiesh et al., 2024). In diabetic patients, cardiac fibrosis is often closely linked to heart failure, loss of contractile function, and decline in cardiac performance. Therefore, NOX-induced activation of the TGF-β/Smad pathway not only promotes fibrosis but also accelerates cardiac remodeling, leading to a vicious cycle of structural damage.

### 3.3 Interaction between NOX and MAPK signaling pathway

The MAPK signaling pathway, including ERK, JNK, and p38MAPK, is a key regulator of cell proliferation, stress response, and apoptosis (Zhang Q. et al., 2022). In DCM, NOX activates members of the MAPK family through ROS generation, triggering cardiomyocyte proliferation, apoptosis, and cardiac remodeling. ROS activate ERK1/2, JNK, and p38MAPK by altering the intracellular redox environment, and these MAPK family members further regulate cell growth, differentiation, and death (Cui et al., 2022).

In the hyperglycemic environment of diabetes, NOX enhances MAPK activity, leading to a stress response in cardiomyocytes, which induces cytokine secretion and results in inflammation in cardiac tissue. NOX5 generates ROS, which activates the MAPK pathway, particularly the p38, JNK, and ERK1/2 pathways, in DCM. Studies show that NOX5 expression enhances the phosphorylation of these redox-sensitive MAPK pathways, further promoting myocardial hypertrophy, fibrosis, and cardiac dysfunction in DCM, worsening the pathological changes in the heart (Zhao et al., 2020). The interaction between ROS and the MAPK signaling pathway in DCM remains an area for further research. However, current studies have confirmed that NOX, through ROS, regulates the MAPK pathway and plays a pivotal role in cardiac remodeling.

### 3.4 Interaction between NOX and PI3K/Akt signaling pathway

In the pathogenesis of DCM, the PI3K/Akt signaling pathway is a key regulator of cell survival and anti-apoptotic responses, and its activity is strongly affected by oxidative stress (Ren et al., 2020). NOX influences PI3K/Akt pathway activation through ROS generation, regulating cardiomyocyte survival and anti-apoptotic responses. On one hand, moderate ROS levels can activate the PI3K/Akt pathway, promoting cardiomyocyte survival. On the other hand, under high glucose conditions, NOX upregulation induces ROS overload, which may lead to abnormal activation or depletion of the PI3K/Akt pathway, thereby exacerbating cell apoptosis, myocardial injury, and structural remodeling (Liu and Zhang, 2015). Thus, the interaction between NOX and the PI3K/Akt signaling pathway plays a critical role in cardiac protection and may influence the progression of DCM by regulating the balance of this pathway.

NOX activates several important signaling pathways, such as NF-κB, TGF-β, MAPK, and PI3K/Akt, through ROS generation. These pathways play crucial roles in processes like cardiomyocyte proliferation, apoptosis, and fibrosis. In the following section, we will explore in detail the specific consequences of these signaling pathways in DCM and how they exacerbate cardiac structural and functional changes through inflammatory responses, myocardial fibrosis, and other pathological reactions.

## 4 Downstream effects of OS and their relationship with DCM

Sustained OS in the heart, primarily driven by hyperglycemia, is a well-established contributor to the structural and functional changes observed in DCM. This maladaptive process involves pathological responses such as inflammation and cardiomyocyte apoptosis (Jubaidi et al., 2021). In the diabetic state, excessive production of ROS mediated by NOX serves as a major source of OS. The persistent accumulation of ROS not only directly induces oxidative damage to cardiomyocytes but also activates multiple downstream signaling pathways, resulting in key pathological responses, including inflammation, mitochondrial dysfunction, and myocardial fibrosis. These downstream effects are interconnected and collectively accelerate myocardial dysfunction and structural remodeling (Fiorentino et al., 2013; Zhang et al., 2023).

### 4.1 Inflammatory response

**FIGURE 1:**
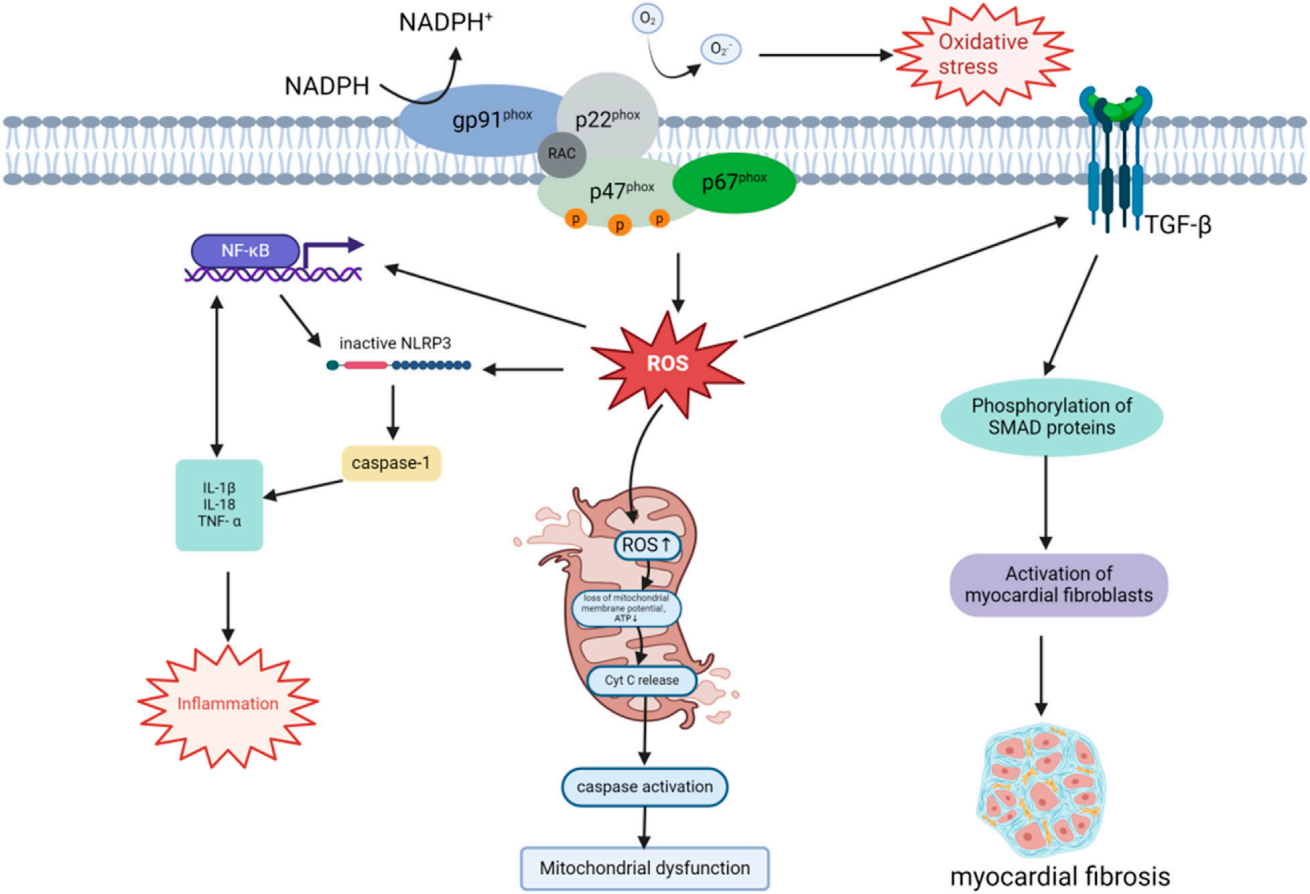
Mechanisms of NOX-derived ROS in DCM progression. The NOX enzyme complex, anchored to the plasma membrane, generates ROS by transferring electrons from NADPH, thereby inducing OS. ROS activate the NF-κB pathway, promote NLRP3 inflammasome assembly, and activate caspase-1, leading to the secretion of pro-inflammatory cytokines such as IL-1β, IL-18, and TNF-α. NLRP3 inflammasome activation further enhances NF-κB signaling through IL-1β release, establishing a positive feedback loop that exacerbates local and systemic inflammation. Additionally, ROS accumulation disrupts mitochondrial membrane potential and ATP synthesis, triggering cytochrome C release and caspase activation, which compromise mitochondrial function and dynamics. ROS also amplify the TGF-β/Smad signaling pathway by enhancing TGF-β receptor activation and downstream Smad protein phosphorylation. This promotes cardiac fibroblast activation and extracellular matrix production, including collagen, driving myocardial remodeling and fibrosis. These interconnected mechanisms collectively contribute to the pathogenesis of DCM.

Inflammation is an adaptive mechanism that helps restore cellular homeostasis under stress (Al-Rasheed et al., 2017; Zhang et al., 2023). However, when stress persists, as in diabetes, this adaptive response becomes maladaptive, resulting in harmful consequences (Jubaidi et al., 2021). Excessive ROS generated by NOX activate the nuclear factor kappa-light-chain-enhancer of activated B cells (NF-κB) pathway and enhance the advanced glycation end-products/receptor for advanced glycation end-products (AGE/RAGE) axis. NF-κB, a redox-sensitive protein complex, plays a pivotal role in inflammation (Shah and Brownlee, 2016). Upon activation, NF-κB promotes the transcription and release of inflammatory mediators such as tumor necrosis factor-alpha (TNF-α), amplifying pro-inflammatory cytokine production and exacerbating myocardial injury (Zhuang et al., 2023). Consequently, NF-κB serves as a key initiator of inflammation in DCM (Figure 1).

NOX-induced OS activates the NLRP3 inflammasome through the excessive production of ROS. The NLRP3 inflammasome is composed of NLRP3, the apoptosis-associated speck-like protein containing a CARD domain (ASC), and pro-caspase-1 (Ding et al., 2019). It plays a critical role in regulating the inflammatory response by activating caspase-1, which facilitates the maturation and secretion of pro-inflammatory cytokines such as IL-1β and IL-18. This process intensifies both local and systemic inflammation (Abais et al., 2015; Yang et al., 2019; Zhang L. et al., 2022; Zhao et al., 2024), contributing to the development and progression of DCM (Figure 1). NF-κB enhances NLRP3 inflammasome expression and amplifies inflammation by upregulating the transcription of the NLRP3 gene. Conversely, NLRP3 inflammasome activation increases NF-κB signaling by promoting the production of cytokines such as IL-1β, thereby establishing a positive feedback loop. These interconnected mechanisms sustain chronic inflammation and accelerate the progression of DCM (Luo et al., 2014; Peng et al., 2022).

### 4.2 Mitochondrial dysfunction

NOX generates excessive ROS, initiating OS within cells and leading to mitochondrial dysfunction (Vásquez-Trincado et al., 2016). NOX enzymes transfer electrons to oxygen molecules via NADPH, producing ROS such as superoxide anion (O_2_^−^), which directly damages proteins and lipids, impairing mitochondrial bioenergetic function. This oxidative damage extends to mitochondrial DNA, resulting in promoter inactivation, downregulation of mitochondrial gene expression, and decreased mitochondrial membrane stability, ultimately disrupting ATP synthesis (Singh et al., 2019). Insufficient mitochondrial energy supply not only impairs cellular function but also exacerbates intracellular redox imbalance, further triggering cell death. ROS accumulation also leads to the loss of mitochondrial membrane potential, a key hallmark of mitochondrial dysfunction, and promotes apoptosis by activating intracellular apoptotic pathways such as cytochrome c release and caspase activation (Rehfeldt et al., 2021). These processes exacerbate myocardial cell death. Chronic OS disrupts mitochondrial fusion and fission, impairing mitochondrial dynamics and destabilizing the mitochondrial network (Bhatti et al., 2017). This dysregulation is particularly pronounced in dilated cardiomyopathy DCM. Prolonged ROS-induced mitochondrial damage ultimately results in cardiac dysfunction, characterized by myocardial hypertrophy, fibrosis, and loss of contractile function, which are hallmark pathological features of DCM (Figure 1).

### 4.3 Myocardial fibrosis

Myocardial fibrosis is a hallmark of cardiac remodeling in DCM (Sung et al., 2015). It occurs alongside hypertrophy as part of the cardiac remodeling process, aiming to preserve the structural integrity of the heart. Fibrosis involves the deposition of fibrotic components, such as collagen I and collagen III, within the extracellular matrix (ECM) to replace dead myocardial cells. However, the stiff nature of collagen tissue reduces the heart’s contractility and flexibility, impairing cardiac function (Frangogiannis, 2021). OS, caused by an imbalance between antioxidant defenses and ROS production, is a central mechanism in DCM and a key regulator of myocardial fibrosis (Zhao et al., 2023). For instance, ROS generated by NOX4 play a pivotal role in promoting myocardial fibrosis through activation of the TGF-β/Smad signaling pathway, which induces excessive collagen deposition and cardiac sclerosis in DCM (Figure 1). In one study, Li and colleagues experimentally demonstrated that NOX4 is a major driver of myocardial OS and fibrosis under diabetic conditions. Diabetes was shown to significantly upregulate NOX4 expression in myocardial tissue, resulting in excessive ROS production. This ROS increase activated the TGF-β/Smad signaling pathway, stimulating cardiac fibroblast activation and promoting collagen deposition, ultimately leading to myocardial fibrosis (Jiang et al., 2014; Li et al., 2019). Furthermore, studies using a prediabetic metabolic syndrome rat model demonstrated that inhibiting OS can effectively reduce myocardial fibrosis (Kusaka et al., 2016).

## 5 Targeting NOX in the treatment of DCM: experimental studies and clinical applications

The downstream pathological mechanisms triggered by OS, including inflammation, mitochondrial dysfunction, and fibrosis (Peng et al., 2022), form a complex network driving the progression of DCM. These mechanisms interact synergistically, progressively impairing myocardial cell function and compromising the structural integrity of the heart. Importantly, OS not only serves as the central driving force behind these mechanisms but also continuously amplifies them. For instance, the inflammatory response stimulates ROS generation via the release of pro-inflammatory factors (Shah and Brownlee, 2016), while mitochondrial dysfunction exacerbates OS through a feedback loop of mitochondrial ROS (Ren et al., 2010; Singh et al., 2019). Additionally, fibrotic tissue stiffening and collagen deposition accelerate myocardial remodeling (Zhao et al., 2023). This intricate pathological network suggests that effective therapeutic intervention in DCM must target upstream mechanisms to block the source of OS. In this regard, NOX has emerged as a central focus for targeted therapy. Compared to xanthine oxidase and mitochondrial respiration, NOX has been identified as a major source of ROS in cardiac tissue (MacCarthy et al., 2001). Excessive activation of NOX not only initiates OS but also regulates multiple pathological signaling pathways through its various isoforms, such as NOX2 and NOX4. In the diabetic state, NOX-driven overproduction of ROS has been confirmed as a critical trigger for inflammation, mitochondrial dysfunction, and fibrosis. Thus, targeting NOX holds significant therapeutic potential, not only by directly reducing ROS generation but also by interrupting the upstream cascade of pathological mechanisms, offering a novel approach to alleviating DCM.

### 5.1 Dapagliflozin

Dapagliflozin is a highly effective, reversible, and selective sodium-glucose co-transporter-2 (SGLT2) inhibitor widely used in the treatment of type 2 diabetes (Dhillon, 2019). While its primary action is in the kidneys, clinical data highlight the cardiovascular benefits of SGLT2 inhibitors, emphasizing their potential to prevent cardiovascular events and heart failure (Arow et al., 2020). Clinical studies have shown that patients receiving dapagliflozin therapy experience a reduced risk of heart failure exacerbation and cardiovascular-related mortality (McMurray et al., 2019). Animal studies further support these findings. In BTBR mice, dapagliflozin slows the progression of DCM, inhibits NLRP3 inflammasome activation and fibrosis, and promotes activation of the mTORC2 pathway in cardiac tissue (Ye et al., 2017; Chen et al., 2020). Additionally, dapagliflozin exerts cardioprotective effects in diabetic mice under angiotensin II stress by reducing ROS levels and calcium transport activity in membrane channels, thereby decreasing OS and providing antioxidant protection to myocardial cells (Arow et al., 2020).

**FIGURE 2:**
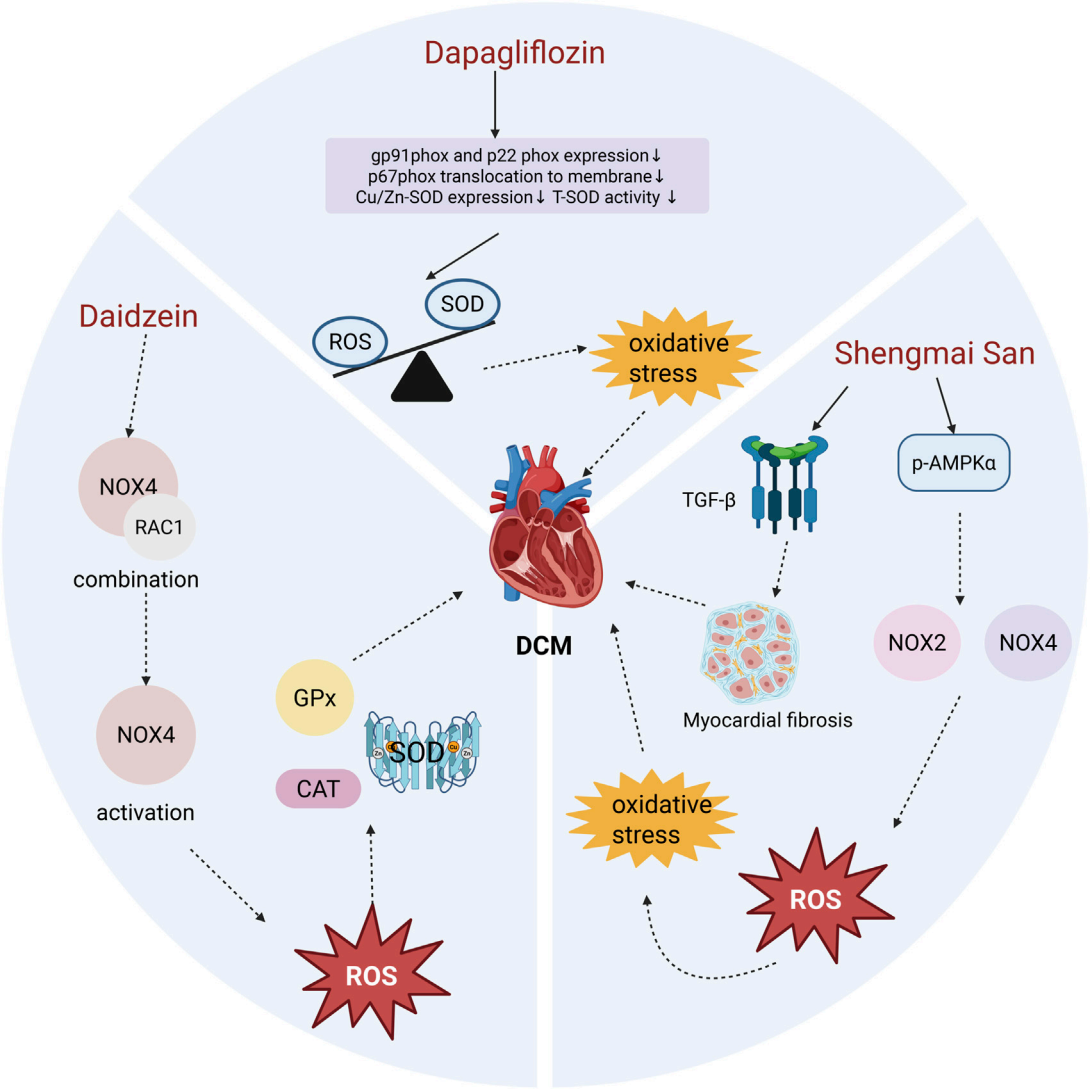
Effects of Dapagliflozin, Daidzein, and Shengmai San on Myocardial Oxidative Stress in DCM. Dapagliflozin treatment significantly reduced the compensatory increase in T-SOD activity and the expression of Cu/Zn-SOD, without altering the levels of Mn-SOD, GPx, or CAT. Although overall SOD levels decreased following Dapagliflozin treatment, it effectively suppressed the production of superoxide anions, restoring the balance between oxidants and antioxidants and improving myocardial OS. Daidzein prevents the interaction between RAC1 and NOX4, thereby inhibiting NOX4 activation. This reduction in NOX4 activity lowers ROS production, maintains antioxidant enzyme activity, and alleviates DCM. Shengmai San enhances the phosphorylation of AMPKα (p-AMPKα), which inhibits the expression and activity of NOX2 and NOX4. This leads to reduced ROS generation and mitigates myocardial OS and fibrosis.

In a study by Xing et al., experimental results demonstrated that dapagliflozin protects myocardial cells from hyperglycemia-induced damage by inhibiting NOX-mediated OS (Xing et al., 2021). Chen et al. further confirmed that dapagliflozin reduces the levels of NOX subunits p67phox and NOX4, preventing ROS accumulation (Chen et al., 2019). Specifically, dapagliflozin treatment decreased the expression of gp91phox and p22phox in the membrane NOX complex and inhibited the translocation of the p67^phox subunit to the membrane. Although superoxide dismutase (SOD) levels were reduced following dapagliflozin treatment, the production of superoxide anions was significantly suppressed (Xing et al., 2021). These findings suggest that dapagliflozin inhibits the recruitment of cytosolic p67phox and reduces its binding to p22phox, suppressing activation of the gp91phox isoform and correcting the imbalance between oxidants and antioxidants, thereby improving myocardial OS (Figure 2). While further exploration of the underlying mechanisms is needed, the existing evidence indicates that dapagliflozin holds promise as a targeted NOX therapy for DCM.

The pharmacological profile of dapagliflozin indicates that it is rapidly absorbed from the gastrointestinal tract following oral administration, typically reaching peak plasma concentrations within 2 h under fasting conditions. Its bioavailability is approximately 78%, meaning the majority of the drug enters the bloodstream after oral administration (Anderson, 2014). The average steady-state volume of distribution is 118 L, with approximately 91% of the drug bound to plasma proteins. Dapagliflozin is primarily excreted via urine (approximately 75%), mainly as the parent compound and metabolites, with the remaining 21% eliminated through feces. After a single 10 mg dose in healthy subjects, the mean terminal elimination half-life of dapagliflozin is approximately 12.9 h (Dhillon, 2019). In patients with type 2 diabetes, dapagliflozin is generally well-tolerated over extended periods (Jabbour et al., 2018). These pharmacokinetic characteristics make it effective in maintaining therapeutic effects and convenient for daily management. While its safety profile is generally favorable, adverse effects and contraindications remain, including urinary tract infections, hypoglycemia, hypotension, and diabetic ketoacidosis (Plosker, 2014). Dapagliflozin has demonstrated significant cardiovascular protection in patients with heart failure and diabetes. However, the specific mechanisms and long-term effects of this action in diverse patient populations require further investigation.

### 5.2 Daidzein

Daidzein is a naturally occurring plant-derived estrogen, classified as a non-steroidal estrogen, with diverse pharmacological properties, including anti-hemolytic, antioxidant, and anti-inflammatory effects (Dwiecki et al., 2009; Zhou D. D. et al., 2021). Ankit P. Laddha and colleagues investigated the effects of daidzein in a rat model of DCM and found that daidzein treatment decreased oxidative damage to cardiomyocytes by maintaining AMPK and SIRT1 levels (Laddha and Kulkarni, 2021). Furthermore, daidzein treatment prevented necrotic damage in cardiac tissue and inhibited myocardial fibrosis. Notably, compared to normal control animals, the diabetic control group exhibited significantly increased expression of NOX4 and RAC1, which was markedly reduced following daidzein treatment (Laddha and Kulkarni, 2021).

NOX4 consists of transmembrane and cytosolic subunits. Under hyperglycemic conditions, the cytosolic subunit becomes phosphorylated and associates with the membrane subunit to form a functional oxidase complex. This complex transfers electrons from NADPH to oxygen, generating superoxide radicals that promote OS and cellular damage. Activation of the NOX4 complex also depends on the small GTPase RAC1, which exists in the cytoplasm as a dimer with Rho GDP-dissociation inhibitor (Rho-GDI). When RAC1 binds to GTP, it associates with the NOX4 membrane subunit, further activating the complex and generating ROS (Gorin et al., 2003; Acevedo and González-Billault, 2018). Studies have demonstrated that flavonoids inhibit RAC1 expression and prevent its binding to the NOX4 membrane subunit, thereby preventing NOX4 activation and alleviating OS. This inhibition also stabilizes antioxidant enzyme levels, including superoxide dismutase (SOD), glutathione peroxidase (GPx), and catalase (CAT) (Figure 2). Thus, daidzein alleviates the progression of DCM primarily by inhibiting NOX4-induced OS in cardiac tissue, making it a promising therapeutic approach for DCM.

Daidzein shows a small plasma peak approximately 1 h after intake, indicating its initial absorption in the small intestine. A larger peak appears 5–8 h post-ingestion, reflecting the recirculation of conjugates and absorption in the colon. Plasma concentration reaches its maximum around 7 h after ingestion. Nearly all of the daidzein is rapidly absorbed and metabolized, with minimal excretion in feces and urine, although up to 30% of the ingested daidzein can be recovered in urine (Elksnis et al., 2023). In terms of bioavailability, studies have shown that daidzein in its glucoside form has higher bioavailability compared to its aglycone form (Rüfer et al., 2008). Additionally, insoluble fibers such as inulin have been found to enhance the absorption of soybean isoflavones (Piazza et al., 2007). These findings provide insights into optimizing therapeutic applications of daidzein. However, further clinical trials are needed to evaluate potential adverse effects and ensure safety. Common side effects include gastrointestinal discomfort, sore throat, weight gain, rash, and breast tenderness. In rare cases, vaginal bleeding has been reported 2–3 days after intake during the third year post-menopause (Akhlaghipour et al., 2024). These side effects underscore the importance of careful consideration in the clinical use of daidzein, particularly for specific patient populations.

### 5.3 Shengmai San

Shengmai San (SMS) is a traditional Chinese medicine formula composed of Panax ginseng (Araliaceae, promotes Qi and disperses stagnation), Ophiopogon japonicus (Liliaceae, nourishes Yin and generates fluids), and Schisandra chinensis (Schisandraceae, tonifies Qi) (Ma et al., 2016; Ouyang et al., 2022; Wang et al., 2022). Clinically, SMS is considered a typical formula for tonifying Qi and nourishing Yin, traditionally used to treat ischemic diseases and diabetes, primarily by targeting OS and inflammation (Mo et al., 2015). Recent evidence suggests that SMS has significant protective effects on the heart and vasculature. Studies by Zhao et al. demonstrated that SMS alleviates cardiac hypertrophy and fibrosis through the TGF-β-dependent pathway (Zhao et al., 2016) and exhibits anti-myocardial fibrosis effects in high-fat diet and streptozotocin (STZ)-induced rat models (Ni et al., 2011). Additionally, SMS possesses antioxidant and anti-inflammatory properties and acts as a regulator of lipid metabolism (Li et al., 2013). SMS also mitigates myocardial oxidative damage by activating AMPKα and inhibiting NOX signaling, showing therapeutic potential in treating DCM (Lu et al., 2021).

As a traditional herbal compound, SMS intervenes in NOX-mediated OS through multiple pathways. It reverses the elevation of cardiac NOX2 and NOX4 protein levels while inhibiting the translocation of p47phox and p67phox to the cell membrane. Moreover, ginsenoside Rg1, an active component of Panax ginseng, has been shown to downregulate NOX2 and reduce ROS production in hippocampal neurons treated with H_2_O_2_ (Xu et al., 2019). Furthermore, AMPK plays a pivotal antioxidant role in the cardiovascular system by inhibiting NOX expression and activity (Li et al., 2020). Experimental results indicate that SMS significantly enhances p-AMPKα protein levels in diabetic hearts (Tian et al., 2018; Lu et al., 2021) (Figure 2). In conclusion, SMS enhances overall antioxidant capacity, effectively alleviates OS damage in DCM, and demonstrates its potential as a promising therapeutic strategy for this condition.

With the expansion of clinical applications, SMS has been developed into various formulations, including Shengmai oral liquid, Shengmai capsules, Shengmai granules, Shengmai injection, and Dengzhan Shengmai capsules (Ouyang et al., 2022). The Schisandra lignans in Shengmai granules are rapidly absorbed and cleared in both volunteers and mice, with an absorption half-life of 0.03–0.04 h and a clearance half-life of 0.86–0.88 h. Studies on SMI revealed that 11 components, including ginsenosides and Schisandra lignans, are rapidly distributed to various tissues in rat serum. Schisandra lignans quickly accumulate in tissues, while Ophiopogon saponin D is cleared within 4 h. Ginsenosides Rg1, Re, Rf, and Rg2 are excreted within 8 h, whereas Rb1, Rd, and Rc remain at higher concentrations for up to 96 h (Ouyang et al., 2022). In China, Panax ginseng, Ophiopogon japonicus, and Schisandra chinensis have long been used as raw materials for health products, with well-established safety profiles. Except for Shengmai injection, there have been no reports of clinical toxicity or side effects associated with the oral Shengmai San compound. However, careful attention should be given to dosage and potential interactions with other medications.

A detailed discussion of the pharmacological properties of these three drugs (Dapagliflozin, Daidzein, and Shengmai San) is provided above. Additional drugs targeting NOX to alleviate oxidative stress and aid in the treatment of DCM are summarized in Table 1.

**TABLE 1:** The following are recent reports on related potential drugs.

Drugs	Mechanism	References
Dapagliflozin	Dapagliflozin administration significantly reduced the expression of the membrane-bound NOX subunits gp91phox and p22phox, inhibited the translocation of the p67phox subunit to the membrane, and decreased the protein expression and total activity of compensatorily elevated copper/zinc superoxide dismutase (Cu/Zn-SOD) both *in vivo* and *in vitro*	Xing et al. (2021)
Daidzein	Daidzein alleviate the progression of DCM by inhibiting NOX-4-induced OS in cardiac tissue	Laddha and Kulkarni (2021)
Shengmai San	SMS exerts therapeutic properties against DCM by alleviating myocardial oxidative damage through the activation of AMPKα and the inhibition of NOX signaling	Lu et al. (2021)
Apocynin	Apocynin, as an NOX inhibitor, reduces NOX activity, ROS production, myocardial hypertrophy, fibrosis, and inflammatory responses in diabetic hearts, ultimately improving myocardial function in DCM.	Li et al. (2010)
Diphenyleneiodonium (DPI)	DPI inhibits NOX, reduces the activation of its subunits (such as gp91^phox, p47^phox), decreases ROS production, alleviates OS, and improves cardiac structure and function in diabetes	Privratsky et al. (2003), Zhao et al. (2017)
GKT137831	GKT137831, as a NOX4 inhibitor, may provide a new approach for the treatment of DCM by reducing OS and improving myocardial cell function	Wang et al. (2023)
Cocoa-Carob Blend	Effectively alleviating cardiac OS by reducing the expression of NOX2 and NOX4 and preventing the generation of ROS and oxidative damage in the left ventricle of diabetic animals	García-Díez et al. (2022)
miR-29c	Overexpression of miR-29c downregulates hyperglycemia-induced TNF-α levels, ROS production, and increased NOX activity, thereby alleviating hyperglycemia-induced inflammation and ROS generation	Zhong et al. (2024)
Inhibition or genetic knockout of ASMase	ASMase increases ROS production by stimulating NOX4 expression, thereby triggering cell apoptosis. This effect is completely blocked by an ASMase inhibitor or ASMase knockout. *In vivo* experiments further demonstrate that ASMase deficiency can reverse heart disease induced by a high-fat diet (HFD) in mice, resulting in improved cardiac function, reduced hypertrophy, fibrosis, and apoptosis	Liu et al. (2023)
polydatin	Inhibition of NOX and NF-κB activity exerts cardioprotective effects against myocardial injury induced by hyperglycemia	Tan et al. (2020)
LIPUS	Inhibition of ACE-mediated NOX4-related OS and NLRP3 inflammasome activation in cardiac fibroblasts improves diabetic cardiac fibrosis, thereby providing a potential therapeutic approach for treating DCM.	Weng et al. (2022)
Glucose-dependent insulinotropic polypeptide	GIP may inhibit the expression of the receptor for advanced glycation end-products (RAGE) gene in myocardial cells under hyperglycemic conditions, thereby suppressing the harmful effects of AGEs on myocardial cells. This process is mediated by the inhibition of NOX activity, which is induced by cAMP.	Hiromura et al. (2021)
Inhibition of KLF5	KLF5 induces OS by directly binding to the promoter of NOX4 and upregulating NOX4 expression. This process is accompanied by the accumulation of ceramide in the heart. Pharmacological or genetic inhibition of KLF5 reduces superoxide formation in diabetic mice, prevents ceramide accumulation, and improves cardiac function	Kyriazis et al. (2021)
Inhibition of galectin-3	Inhibition of galectin-3 reduces myocardial injury and cell apoptosis in STZ-induced DCM rats by suppressing the NOX/ROS pathway. This alleviates OS damage, thereby protecting the heart from the detrimental effects of DCM.	Sun et al. (2020)
Silencing HMGCS2 and PPARα	Silencing HMGCS2 and PPARα inhibits NOX activity by reducing ketone body production and the metabolic burden of fatty acids. This results in a decrease in ROS production, alleviates OS, and ultimately mitigates myocardial injury and dysfunction in DCM.	Wang et al. (2020)
Trimetazidine (TMZ)	TMZ treatment improves structural and functional alterations associated with diabetes by inhibiting Nox2 and TRPC3, without affecting glucose, insulin, or AGE levels	Tang et al. (2019)
Andrographolide (Andro)	Andro treatment inhibits cardiac inflammation and OS in a dose-dependent manner, while reducing cardiac cell apoptosis, leading to improvements in cardiac fibrosis and hypertrophy. Additionally, Andro prevents high glucose-induced ROS production by inhibiting NOX activation and enhancing the *in vitro* and *in vivo* expression of nuclear factor erythroid 2-related factor 2 (Nrf2)	Liang et al. (2018)
silencing salusin-β	Silencing salusin-β reduces NOX2 expression and ROS production, which in turn alleviates OS, NFκB activation, and inflammation, thereby mitigating cardiac dysfunction in DCM	Zhao et al. (2017)

Most existing treatment strategies regulate NOX activity through indirect pathways, which can somewhat alleviate the pathological manifestations of DCM. However, due to the limited research on the direct inhibition of NOX, it remains uncertain whether NOX is a core therapeutic target in DCM. Therefore, future research should focus on investigating and validating the direct effects of NOX inhibitors in DCM treatment, and determine whether NOX can serve as a more targeted therapeutic target.

Previous studies have demonstrated that direct NOX inhibitors, such as Apocynin and DPI, can significantly reduce the pathological damage and improve cardiac function in animal models of DCM (Privratsky et al., 2003; Li et al., 2010). Compounds such as Apocynin and DPI are widely used as research tools to inhibit NADPH oxidase activity. These findings provide substantial evidence supporting the role of NOX as a potential target for DCM treatment, suggesting that NOX may play a critical role in the improvement of diabetic cardiomyopathy. Future studies should focus on further evaluating the clinical efficacy of direct NOX inhibition and compare these results with existing therapeutic strategies to more definitively assess the potential of NOX in DCM treatment.

Therefore, the potential and application prospects of NOX as a therapeutic target for DCM clearly warrant further investigation. Direct research targeting NOX could clarify its unique role in DCM treatment, thus enhancing treatment precision and efficacy, and advancing the clinical application of NOX-targeted therapeutic strategies.

## 6 Outlook and conclusion

DCM is one of the most common and severe cardiovascular complications of diabetes. With the increasing number of diabetic patients, DCM has become a leading cause of heart failure and death. Its development is closely associated with chronic oxidative stress, where the overproduction of ROS and the imbalance of the antioxidant defense system are key contributors to the progression of DCM. In recent years, the NOX family, as a primary source of ROS, has been identified as playing a pivotal role in the pathogenesis of DCM. Overactivation of NOX not only directly damages cardiomyocytes but also contributes to inflammation, myocardial fibrosis, and apoptosis through multiple signaling pathways, further exacerbating structural and functional changes in the heart.

Current research suggests that NOX-targeted therapies hold significant promise for reducing oxidative stress and improving heart function. In particular, targeting specific NOX isoforms may provide more precise drug interventions for treating diabetic cardiomyopathy. However, despite preliminary studies showing the efficacy of NOX inhibitors in animal models, translating these findings into clinically applicable therapies remains a significant challenge. Future research should focus on the selective targeting of NOX isoforms, the selectivity of drugs, and their safety profiles to develop NOX inhibitors with clinical potential, particularly for early diagnosis and intervention in diabetic cardiomyopathy.

Moreover, with the advancement of big data, genomics, and proteomics technologies, personalized treatment will become an important direction in managing diabetic cardiomyopathy. By precisely identifying individual variations in NOX activity, ROS levels, and associated signaling pathways, more personalized treatment plans can be developed, maximizing therapeutic outcomes while minimizing side effects.

Although traditional antioxidants play a role in reducing oxidative stress, their clinical effectiveness is limited, often accompanied by side effects. Therefore, future research should prioritize the development of novel antioxidants, particularly those that specifically regulate NOX activity. Additionally, combining NOX inhibitors with existing anti-inflammatory and anti-fibrotic drugs could significantly enhance treatment efficacy for diabetic cardiomyopathy.

NOX represents a critical target for therapeutic intervention in diabetic cardiomyopathy, offering substantial clinical research value. By deepening our understanding of NOX-mediated oxidative stress and its role in DCM, more precise and effective therapeutic strategies are expected to be developed, providing new solutions for the early diagnosis, intervention, and treatment of diabetic cardiomyopathy.
